# Knowledge Translation Interventions to Address Gaps in Rectal Cancer Care

**DOI:** 10.1001/jamanetworkopen.2024.61047

**Published:** 2025-02-17

**Authors:** Erin D. Kennedy, Amandeep Pooni, Selina Schmocker, Carl Brown, Anthony MacLean, Nancy N. Baxter, Lara Williams, Marko Simunovic, Sender Liberman, Sébastien Drolet, Katerina Neumann, Kartik Jhaveri, Richard Kirsch

**Affiliations:** 1 Department of Surgery, Mount Sinai Hospital, Toronto, Ontario, Canada; 2 University of Toronto, Toronto, Ontario, Canada; 3 Zane Cohen Centre for Digestive Diseases, Mount Sinai Hospital, Toronto, Ontario, Canada; 4 Department of Surgery, Toronto East Health Network, Toronto, Ontario, Canada; 5 Department of Colorectal Surgery, St Paul’s Hospital, Faculty of Medicine, University of British Columbia, Vancouver, British Columbia, Canada; 6 Department of Surgery, Cumming School of Medicine, University of Calgary, Calgary, Alberta, Canada; 7 Faculty of Medicine and Health, University of Sydney, Sydney, New South Wales, Australia; 8 Department of Surgery, The Ottawa Hospital, Ottawa, Ontario, Canada; 9 Department of Surgery, McMaster University, St Joseph’s Healthcare, Hamilton, Ontario, Canada; 10 Department of Surgery, McGill University, Montreal, Quebec, Canada; 11 Department of Surgery, Université Laval, Quebec City, Quebec, Canada; 12 Department of Surgery, Queen Elizabeth II Health Sciences Centre, Halifax, Nova Scotia, Canada; 13 Joint Department of Medical Imaging, University Health Network, Mount Sinai Hospital, and Women’s College Hospital, Toronto, Ontario, Canada; 14 Department of Pathology and Laboratory Medicine, Mount Sinai Hospital, Toronto, Ontario, Canada

## Abstract

**Importance:**

Over the last 2 decades, increasing use of multimodal strategies has led to significant improvements in oncologic outcomes for patients with rectal cancer. However, uptake of these strategies varies among centers, suggesting that best evidence is not always implemented into practice.

**Objectives:**

To identify gaps in care and initiate knowledge translation interventions to close existing gaps.

**Design, Setting, and Participants:**

This 3-year multifaceted, prospective quality improvement study was conducted at 8 high-volume rectal cancer centers across Canada. From April 2016 to December 2018, patients with stage I to III rectal cancer undergoing total mesorectal excision were enrolled. Data were analyzed from January 2022 through December 2023.

**Interventions:**

Process measures for multimodal strategies to optimize rectal cancer care were selected and prospectively collected for patients with stage I to III rectal cancer undergoing total mesorectal excision. Knowledge translation interventions were implemented to increase uptake of these strategies.

**Main Outcome and Measure:**

Change in uptake of process measures over the study period, with measures taken every 3 months, from time 1 (baseline) to time 7 (18 months).

**Results:**

Among 645 patients with stage I to III rectal cancer (389 male [60.3%]; mean [SD] age, 68.1 [8.2] years), iterative results showed that uptake of 6 of 12 process measures (eg, presentation at multidisciplinary cancer conference: 22 of 77 patients [28.6%] at time 1 to 64 of 91 patients [70.3%] at time 7; *P* < .001) and 1 pathology measure (inadequate lymph node retrieval: 15 of 77 patients at time 1 [19.5%] to 6 of 91 patients at time 7 [6.6%]; *P* = .002) improved over time. Positive circumferential resection margin, positive distal margin, and inadequate lymph node retrieval rates at 2 years were 44 patients (6.8%), 10 patients (1.6%), and 79 patients (12.2%), respectively.

**Conclusions and Relevance:**

In this study, there was an improvement in 6 process measures and 1 pathology measure for patients with stage I to III rectal cancer. Furthermore, this study led to standardized processes of care for rectal cancer that may facilitate continuous quality improvement and multicenter trials across Canada.

## Key Points

**Question:**

What are the gaps in care for patients with stage I to III rectal cancer, and how can these existing gaps be closed?

**Findings:**

This quality improvement study among 645 patients with stage I to III rectal cancer implemented knowledge translation interventions that were associated with improvements in 6 process measures and 1 pathology measure.

**Meaning:**

This study found that knowledge translation interventions were associated with standardization of processes in high-volume colorectal centers across Canada for continuous quality improvement.

## Teaser

This quality improvement study investigates the association of knowledge translation interventions with closing of gaps in care for patients with rectal cancer in Canada.

## Introduction

Over the last 2 decades, the increasing use of multimodal strategies for treatment of rectal cancer has resulted in significant improvements in patient outcomes. There has been widespread adoption of total mesorectal excision (TME) and greater use of preoperative chemoradiotherapy.^1,2,3,4,5,6^ There has also been increasing use of pretreatment staging with pelvic magnetic resonance imaging (MRI) that has led to more appropriate use of preoperative chemoradiotherapy and has assisted with surgical planning by predicting the status of the circumferential margin.^7,8^ Similarly, there has been increasing use of pathologic assessment using the Quirke method and reporting of the status of the CRM and completeness of the TME, both important quality and prognostic indicators.^9,10^ Finally, multidisciplinary cancer conference (MCC) has enhanced interdisciplinary communication to coordinate more tailored, evidence-based treatment for patients.^2,6,11^

While these multimodal strategies are recommended in published guidelines and are mandatory components of the US Commission on Cancer National Accreditation Program for Rectal Cancer,^5,12,13,14^ the implementation of these strategies varies considerably across centers in North America and Europe.^15,16,17,18,19,20^ This unwarranted variation in the uptake of these strategies suggests that the best evidence is not always implemented into clinical practice and represents a significant quality gap for patients and clinicians.^21^ Closing these quality gaps is important because increasing compliance with these multimodal strategies is associated with improved survival.^21,22^ Furthermore, there has been little research to date on the association of knowledge translation (KT) interventions with reduced variation and closing of quality gaps to optimize the care of patients with rectal cancer. Therefore, the objectives of this study were to identify existing gaps in care and implement KT interventions to close these existing gaps for patients with stage I to III rectal cancer.

## Methods

### Study Design

This was a multifaceted, prospective knowledge-translation quality improvement study conducted at 8 high-volume rectal cancer centers across Canada from April 1, 2016, to December 31, 2018. The study consisted of a 1-year planning phase and a 2-year recruitment phase. The full study protocol has been previously published.^23^ Prior to the start of the study, research ethics approval was obtained from the Mount Sinai Hospital Research Ethics Board (REB) and REBs from all participating centers (the Capital Health REB, Centre Hospitalier Universitaire de Québec REB, McGill University Health Center–Montreal General Hospital REB, University of British Columbia Providence Health Care REB, Unity Health [formerly St. Michael’s Hospital] REB, University of Calgary Conjoint Health REB, and University of Manitoba Bannatyne Campus Health REB). Data sharing agreements were also obtained from all participating centers. Informed consent was waived by research ethics boards at all sites given that this was a quality improvement project that did not directly affect patient care and no personal identifying information was required. The Standards for Quality Improvement Reporting Excellence (SQUIRE) reporting guideline was followed.

### Conceptual Framework

The conceptual framework that guided this study was the Canadian Institute of Health Research Knowledge to Action (KTA).^24^ KTA is the exchange of knowledge between relevant stakeholders that results in action and involves knowledge creation and the action cycle. Knowledge creation involves knowledge inquiry, synthesis, and tools or products, and the action cycle represents activities that may be needed for knowledge application. The KTA process is complex and dynamic, and boundaries between the 2 phases are fluid and permeable. For clarity, study methods are presented in the following sections according to individual components of the KTA cycle.

#### Identify the Problem

Prior to the start of the study, we conducted a survey of 8 centers with representatives who had agreed to participate in the study to inquire about the current processes used at their center.^23^ Results of this survey showed that only 2 centers routinely presented patients with a new diagnosis of rectal cancer at MCC, and only 1 center formally documented this MCC decision in the patient electronic health record. Although all centers reported routine use of MRI for local staging of rectal cancer, only 2 centers reported using a standard MRI protocol and synoptic MRI report. Furthermore, most centers had not formally implemented pathologic assessment using the Quirke method, and there was variable use of the College of American Pathologists (CAP) checklist. No centers had implemented all multimodal strategies, and centers that implemented some strategies seemed to have done so without complete fidelity to best evidence. Therefore, results of this survey suggested that there were existing gaps in care that represented an opportunity to optimize the use of these strategies and processes of care across participating centers.^23^

#### Adapt Knowledge, Assess Barriers, and Tailor to the Local Context

After surveys were completed, our investigative team held an in-person workshop. We invited a surgeon, radiologist, radiation oncologist, medical oncologist, and pathologist from each site that agreed to participate in the study (E.D.K., A.P., C.B., A.M., N.N.B., L.W., M.S., S.L., S.D., K.N., K.J., and R.K.). At the workshop, the investigative team and invited physicians reviewed the current literature; a modified Delphi method was conducted to select relevant process measures to evaluate the uptake of multimodal strategies, including pretreatment MRI, MCC, appropriate use of radiotherapy, TME, and pathologic assessment using the Quirke method.^25^ In addition to selecting process measures, the group identified preexisting tools that had been successfully adopted at each participating center to help facilitate the capture of process measures.^25^

After the workshop, physicians at the participating centers were responsible to launch the study locally at their own sites. At the launch, physicians were encouraged to obtain feedback about facilitators and barriers to implementation of multimodal strategies from their colleagues. Although the specific process measures were not modifiable, physicians were encouraged to modify recommended tools consistent with best practice to facilitate implementation at their specific centers based on the feedback obtained from their colleagues.

#### Knowledge Translation Plan

KT interventions implemented in this study were (1) a multidisciplinary community of practice (CoP) and (2) audit and feedback. The rationale for the selection of these KT interventions has been previously described.^23,26,27,28^

The CoP consisted of the investigative team and physicians participating in the workshop. This was a multidisciplinary CoP that shared best practices with one another about the multimodal processes of care for rectal cancer and acted as local champions for the study at their respective institutions.

Audit and feedback reporting for uptake of the selected process measures was provided to the investigative team and CoP every 3 months for a total of 7 cycles (ie, from time 1 [baseline] to time 7 [18 months]). Data included the uptake of process measures overall for all centers and by site. After each audit and feedback cycle, the investigative team and CoP participated in a teleconference to evaluate progress, identify barriers to success, and discuss strategies to overcome these barriers. Each site was encouraged to identify gaps in care and work together with local colleagues and other CoP members at that site to develop local strategies to close these gaps in care. After the first year of recruitment, a second, in-person workshop was conducted with the CoP. At this workshop, top-performing hospitals were asked to share their practice and implementation strategy with the CoP.

#### Study Outcomes and Evaluation

##### Study Group

Over a 2-year period, patients with stage I to III biopsy-proven adenocarcinoma located 15 cm or less from the anal verge undergoing curative-intent TME surgery at participating sites were enrolled in the study by their treating surgeon. Patients presenting with stage IV or metastatic disease were excluded.

##### Study Outcomes

During the 2-year recruitment phase, we measured the uptake of the 12 process measures. Pathologic measures were also collected to ensure appropriate staging. These also included the rate of positive circumferential margin, defined as tumor cells less than 1 mm from the resection margin, the completeness of the TME, and the number of lymph nodes retrieved. After the second iteration of the audit and feedback, the CoP decided to also collect oncologic outcomes, including rate of local recurrence, disease-free survival, and overall survival.

##### Data Collection

A dedicated research coordinator at each site prospectively collected process and pathology measures and data for oncologic outcomes. Data were entered into a web-accessible database designed for the study. All research coordinators (including S.S.) participated in a 2-hour orientation session, including review of a data dictionary developed for the study that contained definitions of all study measures. Source documentation for MCC, MRI, and operating room (OR) and pathology reports was obtained, and central review was completed to ensure the completeness and accuracy of data.

### Statistical Analysis

We principally used descriptive statistics for all collected measures. Among continuous data, normally distributed variables were presented as means with SDs and nonnormally distributed variables as medians with IQRs. Counts and percentages were used to present categorical data. For the primary analysis, change in uptake of process measures over the study period was evaluated using a Cochrane-Armitage trend test. Multivariable logistic regression models were fitted for all process measures with 2-sided *P* < .05 to evaluate the association with time adjusting for site and pathologic stage. Secondary analysis included pathology measures, which were evaluated for overall results and change over time. Patient oncologic outcomes were reported as rates using all available data and included local recurrence, distant metastasis, and disease-free survival at 2 years. Kaplan-Meier curves for these outcomes were generated. All analyses were conducted using SAS statistical software version 9.4 (SAS Institute). For both primary and secondary analysis, missing data were handled by deletion. Data were analyzed from January 2022 through December 2023.

## Results

### Patient, Tumor, and Treatment Characteristics

A total of 645 patients (389 male [60.3%]; mean [SD] age, 68.1 [8.2] years) were enrolled April 1, 2016, to December 31, 2018. Patient, tumor, and treatment characteristics are reported in Table 1. Pretreatment MRI was performed in 552 patients (85.6%), and 474 of 572 patients with data (82.9%) had mid- to low-rectal cancer. A total of 348 patients (54.0%) received neoadjuvant chemoradiotherapy, while 389 surgical procedures (60.3%) were performed laparoscopically and 450 of 632 procedures with data (71.2%) avoided a permanent stoma. The final pathology was that most patients had stage II or III cancer, with T2 or T3 disease among 438 of 624 patients with data (70.1%) and node-positive disease among 216 of 620 patients with data (34.8%). There were 193 patients (30.0%) who received adjuvant chemotherapy. Overall, there were 77 patients at time 1, 110 patients at time 2, 90 patients at time 3, 102 patients at time 4, 86 patients at time 5, 89 patients at time 6, and 91 patients at time 7 (Table 2).

**Table 1.** Patient, Tumor, and Treatment Characteristics

Characteristic	Patients, No. (%) (N = 645)
Age, mean (SD), y	68.1 (8.2)
Sex	
Male	389 (60.3)
Female	256 (39.7)
Site	
1	130 (20.2)
2	48 (7.4)
3	92 (14.3)
4	63 (9.8)
5	51 (7.9)
6	154 (23.9)
7	54 (8.4)
8	53 (8.2)
Tumor height, cm	
High (10.1-15.0)	98 (17.1)
Mid (5.1-10.0)	275 (48.1)
Low (0-5.0)	199 (34.8)
Receipt of chemoradiotherapy	348 (54.0)^a^
Receipt of adjuvant chemotherapy	193 (30.0)
Restorative surgery (n = 632)	450 (71.2)
Laparoscopic approach	389 (60.3)
pT category (n = 624)	
T0	58 (9.2)
T1	94 (15.1)
T2	190 (30.4)
T3	248 (39.7)
T4	34 (5.4)
pN category (n = 620)	
Negative	404 (65.4)
Positive	216 (34.8)
p Stage	
Stage 1	277 (44.3)
Stage 2	132 (21.1)
Stage 3	216 (34.6)

^a^ All of these patients received preoperative chemoradiotherapy.

**Table 2.** Uptake of Process Measures

Process measure	Patients, No. (%)								*P* value
	Overall (N = 645)	Time 1 (n = 77)^a^	Time 2 (n = 110)^a^	Time 3 (n = 90)^a^	Time 4 (n = 102)^a^	Time 5 (n = 86)^a^	Time 6 (n = 89)^a^	Time 7 (n = 91)^a^	
Pretreatment CEA	471 (73.0)	63 (81.8)	73 (66.4)	69 (76.7)	82 (80.4)	68 (79.1)	59 (66.3)	57 (62.6)	.04
Pretreatment CT of chest, abdomen, and pelvis	611 (94.7)	74 (96.1)	105 (95.6)	87 (96.7)	97 (95.1)	85 (98.8)	80 (89.9)	83 (91.2)	.06
MCC presentation	375 (58.1)	22 (28.6)	47 (42.7)	61 (67.8)	51 (50.0)	62 (72.1)	68 (76.4)	64 (70.3)	<.001
MCC synoptic report									
No. with data	375	22	47	61	51	62	68	64	NA
No. (%)	183 (48.8)	7 (31.8)	13 (27.6)	24 (39.3)	25 (49.0)	30 (48.4)	37 (54.4)	47 (73.4)	<.001
Pretreatment MRI	552 (85.6)	64 (83.1)	90 (81.8)	73 (81.1)	93 (91.2)	80 (93.0)	78 (87.6)	74 (81.3)	.34
Synoptic MRI report									
No. with data	555	64	90	74	93	80	80	74	NA
No. (%)	299 (53.8)	29 (45.3)	39 (43.3)	41 (55.4)	44 (47.3)	43 (53.8)	51 (63.4)	52 (70.3)	<.001
Radiation oncology peer review									
No. with data	352	54	54	48	61	51	50	34	NA
No. (%)	118 (33.5)	21 (38.9)	18 (33.3)	19 (39.6)	23 (37.7)	15 (29.4)	13 (26.0)	9 (26.5)	.10
Radiation oncology checklist									
No. with data	349	54	54	48	61	51	49	32	NA
No. (%)	71 (20.3)	11 (20.4)	13 (24.1)	16 (33.3)	11 (18.0)	6 (11.8)	6 (12.2)	8 (25.0)	.20
Preoperative stoma site marking									
No. with data	614	74	100	83	99	82	86	90	NA
No. (%)	465 (75.7)	49 (56.6)	64 (64.0)	69 (83.1)	71 (71.7)	66 (80.5)	72 (83.7)	74 (82.2)	<.001
Synoptic OR report	371 (57.5)	36 (46.8)	46 (41.8)	52 (57.8)	50 (49.0)	61 (70.9)	60 (67.4)	66 (72.5)	<.001
CAP checklist	602 (93.3)	65 (84.4)	103 (93.6)	85 (94.4)	96 (94.1)	81 (94.2)	88 (98.9)	84 (92.3)	.03
Quirke method	473 (73.3)	51 (66.2)	82 (74.6)	77 (85.5)	63 (61.8)	63 (73.3)	67 (75.3)	70 (76.9)	.47

Abbreviations: CAP, College of American Pathologists; CEA, carcinoembryonic antigen; CT, computed tomography; MCC, multidisciplinary cancer conference; MRI, magnetic resonance imaging; NA, not applicable; OR, operating room.

^a^ Measures were taken every 3 months, from time 1 (baseline) to time 7 (18 months).

### Uptake of Process Measures

The overall uptake of the 12 process measures ranged from the lowest, 71 of 349 patients with data for the radiation oncology checklist (20.3%) and 118 of 352 patients with data for radiation oncology peer review (33.5%), to the highest, 611 patients (94.7%) for preoperative computed tomography (CT) imaging (94.7%), followed by 602 patients for the CAP checklist (93.3%) and 552 patients for preoperative MRI (85.6%) (Table 2). Iterative results showed that 6 of 12 process measures improved over time, including presentation at MCC (22 patients [28.6%] at time 1 to 64 patients [70.3%] at time 7; *P* < .001), synoptic MCC report (7 of 22 patients with data [31.8%] at time 1 to 47 of 64 patients with data [73.4%] at time 7; *P* < .001), synoptic MRI report (29 of 64 patients with data [45.3%] at time 1 to 52 of 74 patients with data [70.3%] at time 7; *P* < .001), preoperative stoma marking (49 of 74 patients with data [66.2%] at time 1 to 74 of 90 patients with data [82.2%] at time 7; *P* < .001), synoptic OR report (36 patients [46.8%] at time 1 to 66 patients [72.5%] at time 7; *P* < .001), and CAP checklist (65 patients [84.4%] at time 1 to 84 patients [92.3%] at time 7; *P* = .03) (Table 2). Uptake of 5 of the remaining measures did not change. Of these, pretreatment CT of the chest, abdomen, and pelvis (74 patients [96.1%]) and pretreatment MRI (64 patients [83.1%]) had a high baseline uptake of more than 80%, and the baseline uptake for pathologic assessment with the Quirke method was 51 patients (66.2%). The uptake for pretreatment carcinoembryonic antigen was 63 patients (81.8%) at baseline and significantly worsened over the course the study, to 57 patients at time 7 (62.6%) (*P* = .04). Time remained associated with change for all process measures after adjustment for participating center and pathologic stage. Site-specific uptake for each process measure is shown in the eTable in Supplement 1.

### Pathologic Measures and Outcomes

Pathologic measures are shown in Table 3. Overall rates for positive circumferential margin, positive distal margin, and inadequate lymph node retrieval (ie, <12 lymph nodes) were 44 patients (6.8%), 10 patients (1.6%), and 79 patients (12.2%), respectively. While reporting of TME quality was strongly encouraged, this was not a mandatory component of the CAP checklist and was available for only 495 patients (76.7%). Of these, 38 patients (7.7%) had an incomplete TME specimen. Over time, pathologic measures did not vary, with the exception of an improvement (ie, decrease) in inadequate lymph node retrieval (15 patients at time 1 [19.5%] to 6 patients [6.6%] at time 7; *P* = .002).

**Table 3.** Pathologic Measures and Outcomes

Pathologic outcome	Patients, No. (%)								
	Overall (N = 645)	Time 1 (n = 77)^a^	Time 2 (n = 110)^a^	Time 3 (n = 90)^a^	Time 4 (n = 102)^a^	Time 5 (n = 86)^a^	Time 6 (n = 89)^a^	Time 7 (n = 91)^a^	*P* value
Positive CRM	44 (6.8)	5 (6.5)	7 (6.4)	9 (10.0)	5 (4.9)	6 (7.0)	4 (4.5)	8 (8.8)	.98
Positive distal margin	10 (1.6)	1 (1.3)	1 (0.9)	2 (2.2)	3 (2.9)	0	1 (1.1)	2 (2.2)	.86
Incomplete TME									
No. with data	495	48	80	82	74	70	75	66	NA
No. (%)	38 (7.7)	0	9 (11.3)	9 (11.0)	9 (12.2)	4 (5.7)	1 (1.3)	6 (9.1)	.63
Inadequate lymph node retrieval	79 (12.2)	15 (19.5)	17 (15.5)	9 (10.0)	17 (16.7)	11 (12.8)	4 (4.5)	6 (6.6)	.002

Abbreviations: CRM, circumferential margin; NA, not applicable; TME, total mesorectal excision.

^a^ Measures were taken every 3 months, from time 1 (baseline) to time 7 (18 months).

### Short-Term Oncologic Outcomes

Patient outcomes for local recurrence, distant metastasis, disease-free survival, and overall survival were obtained for 405 patients (62.8% of the cohort), with a median (IQR) follow-up of 2.3 (1.9-2.9) years. Rates of local recurrence, disease-free survival, and overall survival were 10 patients (3.2%), 343 patients (84.7%), and 370 patients (91.4%) respectively. Kaplan-Meier estimates for disease-free survival were 91.5% (95% CI, 88.7%-94.3%) at 1 year and 84.3% (95% CI, 80.5%-88.1%) at 3 years.

## Discussion

For this quality improvement study, we successfully implemented CoP and audit and feedback as KT interventions, which were associated with improvement in the uptake of 6 of 12 process measures and 1 pathology measure and achieved excellent rates of local recurrence and overall survival. The CoP was one of the main reasons for the success of this project given that it promoted active engagement and learning from a multidisciplinary perspective and motivated all members to achieve the study goals. A systematic review on quality improvement collaboratives^29^ showed that this strategy was associated with a median (range) 12% (4%-61%) improvement in quality improvement measures, and the strategy has been widely adopted as an approach to shared learning and improvement in health care.^27,30^ Certainly, one unexpected benefit of the CoP was that over the course of the study, the CoP evolved into a collaborative network of physicians and institutions who have continued to work together beyond the completion of this project.

The use of audit and feedback in this study was effective given that it promoted healthy competition between institutions that was associated with changes in physician behavior, with increased uptake of process measures. The format of our audit and feedback was structured to optimize success by using local data with support of respected, local colleagues and the CoP; repeated iterations of the feedback; and data provided in verbal and written formats.^28^ We also used audit and feedback to identify top performers and requested that top performers share their implementation plans so that other centers could learn and possibly adapt these strategies locally. A Cochrane review of randomized clinical trials (RCTs)^28^ that assessed the effectiveness of audit and feedback to improve patient outcomes reported an absolute median (IQR) risk difference of 0.4% (−1.3% to 1.6%) for dichotomous outcomes and 17% (1.5% to 17%) for continuous outcomes. This review suggested that these results were considered to be small but potentially important improvements in patient outcomes, and those findings validate our own study results.

### Strengths and Limitations

The main strength of this study was that it was a prospective study specifically designed to capture selected measures for rectal cancer. A database was created specifically for the study, and only patients with stage I to III rectal cancer undergoing TME were included. Data were collected by dedicated and trained abstractors, and source documentation was used to ensure the completeness and accuracy of the data. Therefore, the database is highly accurate, with unique patient-level process and outcome data. As part of the study, each center was also encouraged to implement synoptic reports for MRI reports, MCC, operative notes, and pathology as data-collection tools, and at most centers, these tools have been adopted as routine or standard of care. This has led to standardization of the process measures across our centers and created a platform to leverage for grant capture and performing collaborative multicenter trials.

The main limitation of this study was the observational design. Unfortunately, we are not able to definitively conclude that KT interventions, CoP, and audit and feedback led to an increase in the uptake of the process measures beyond secular trends or that the increase in process measures directly led to improvements in patient outcomes, owing to lack of a control group. In addition, the high degree of variation in uptake of process measures across participating sites highlights that there are unknown mechanisms associated with change in the local environment that are not fully understood and are beyond the scope of this study.

With respect to KT interventions, a previous RCT^31^ found no difference in patient outcomes for rectal cancer when high-intensity KT interventions were compared with low-intensity KT interventions on a population level. More recently, a large administrative cohort study^32^ with more than 15 000 patients showed that a high-intensity KT intervention was associated with increased use of preoperative MRI and preoperative radiotherapy compared with a low-intensity KT intervention; however, these increases in process measures were not associated with improvements in patient outcomes. Therefore, future RCTs evaluating KT and other quality improvement interventions and their effect on patient outcomes are important to avoid wasting resources on ineffective interventions. These findings also suggest that new approaches other than traditional KT are likely needed to close quality gaps in care.

Other limitations of this study were that although we had a high level of engagement with radiation oncologists, abstractors had difficulty accessing radiation oncology data given that the electronic health record for radiation oncology at most participating sites was independent from the primary electronic health record used by the hospital. Furthermore, all participating centers were high-volume academic, tertiary centers, and therefore, these results may not be generalizable to other practice settings or fully representative of rectal cancer care in Canada. Additionally, because this study focused on the uptake of process indicators, it is underpowered to detect significant differences for both short- and long-term outcomes.

## Conclusions

In this quality improvement study to optimize rectal cancer care, implementation of KT interventions, CoP, and audit and feedback was associated with improvement in 6 process measures and 1 pathology measure for patients with stage I to III rectal cancer. This study led to standardized processes of care for rectal cancer and has facilitated continuous quality improvement and multicenter trials across Canada. Future RCTs evaluating KT interventions and their effects on patient outcomes are important.

## Notes

Supplement 1. **eTable.** Uptake of Process Measures

Supplement 2.
Data Sharing Statement
