# Supplementary material for: Knowledge Translation Interventions to Address Gaps in Rectal Cancer Care
Source: JAMA Netw Open. 2025 Feb 17;8(2):e2461047. doi: 10.1001/jamanetworkopen.2024.61047 (PMC11833516; doi:10.1001/jamanetworkopen.2024.61047)
Supplement: Supplement 1. — eTable. Uptake of Process Measures [file jamanetwopen-e2461047-s001.pdf]

Supplemental Online Content

Kennedy ED, Pooni A, Schmocker S, et al. Knowledge translation interventions to address gaps in rectal cancer care. *JAMA Netw Open*. 2025;8(2):e2461047. doi:10.1001/jamanetworkopen.2024.61047

eTable. Uptake of Process Measures

This supplemental material has been provided by the authors to give readers additional information about their work.

© 2025 Kennedy ED et al. *JAMA Network Open*.

Supplemental Tables: Uptake of Process Measures

Panel A: Uptake of Process Measures: Pre-treatment CEA

Sites	Overall	Time 1	Time 2	Time 3	Time 4	Time 5	Time 6	Time 7	p-value
**Site 1** (n=130)	85 (65.4)	15(78.9)	15(88.2)	16 (64)	15 (93.8)	13 (68.4)	7 (36.8)	4 (26.7)	**<0.001**
**Site 2** (n=48)	31 (64.6)	9 (90.0)	7 (43.8)	5 (71.4)	2 (100.0)	1 (33.3)	3 (100.0)	4 (57.1)	0.83
**Site 3** (n=92)	61 (66.3)	8 (72.7)	8 (50.0)	4 (80.0)	17 (73.9)	5 (62.5)	9 (75.0)	10 (58.8)	0.97
**Site 4** (n=46)	30 (65.2)	3 (50.0)	7 (77.8)	5 (62.5)	1 (50.0)	2 (50.0)	4 (50.0)	8 (88.9)	0.57
**Site 5** (n=17)	14 (82.4)	1 (100.0)	1 (50.0)	2 (100.0)	3 (75.0)	3 (75.0)	1 (100.0)	3 (100.0)	0.51
**Site 6** (n=51)	50 (98.1)	8 (100.0)	10 (90.9)	17(100.0)	5 (100.0)	4 (100.0)	4 (100.0)	2 (100.0)	0.57
**Site 7** (n=154)	120 (77.9)	7 (87.5)	16 (61.5)	15 (88.2)	20 (83.3)	29 (96.7)	18 (75.0)	15 (60.0)	0.64
**Site 8** (n=54)	36 (66.7)	12 (85.7)	9 (69.2)	2 (50.0)	3 (50.0)	2 (40.0)	5 (62.5)	3 (75.0)	0.24

Panel B: Uptake of Process Measures: Pre-treatment CT CAP

Sites	Overall	Time 1	Time 2	Time 3	Time 4	Time 5	Time 6	Time 7	p-value
**Site 1** (n=130)	119 (91.5)	17 (89.5)	16 (94.2)	24 (96.0)	15 (93.8)	19 (100)	16 (84.2)	12 (80.0)	0.26
**Site 2** (n=48)	44 (91.7)	9 (90.0)	14 (87.5)	7 (100.0)	2 (100.0)	3 (100.0)	2 (66.7)	7 (100.0)	0.71
**Site 3** (n=92)	88 (95.7)	11(100.0)	16 (100.0)	5 (100.0)	22 (95.7)	8 (100.0)	11 (91.7)	15 (88.2)	0.08
**Site 4** (n=46)	45 (97.8)	6 (100.0)	9 (100.0)	8 (100.0)	2 (100.0)	4 (100.0)	7 (87.5)	9 (100.0)	0.70
**Site 5** (n=17)	17 (100.0)	1 (100.0)	2 (100.0)	2 (100.0)	4 (100.0)	4 (100.0)	1 (100.0)	3 (100.0)	-
**Site 6** (n=51)	50 (98.1)	8 (100.0)	11 (100.0)	17(100.0)	5 (100.0)	4 (100.0)	4 (100.0)	1 (50.0)	**0.04**
**Site 7** (n=154)	144 (93.5)	8 (100.0)	24 (92.3)	15 (88.2)	22 (91.7)	29 (96.7)	23 (95.8)	23 (92.0)	0.93
**Site 8** (n=54)	53 (98.2)	14(100.0)	13(100.0)	4(100.0)	6(100.0)	5(100.0)	7(87.5)	4(100.0)	0.22

Panel C: Uptake of Process Measures: MCC Presentation

Sites	Overall	Time 1	Time 2	Time 3	Time 4	Time 5	Time 6	Time 7	p-value
**Site 1** (n=130)	95 (73.1)	3 (15.8)	5 (29.4)	18 (72.0)	16 (100)	19 (100)	19 (100)	15 (100)	**<0.001**
**Site 2** (n=48)	30 (62.5)	1 (10.0)	9 (56.3)	6 (85.7)	1 (50.0)	3 (100.0)	3 (100.0)	7 (100.0)	**<0.001**
**Site 3** (n=92)	7 (7.6)	0 (0.0)	1 (6.3)	1 (20.0)	2 (8.7)	0 (0.0)	1 (8.3)	2 (11.8)	0.45
**Site 4** (n=46)	44 (95.7)	6 (100.0)	8 (88.9)	7 (87.5)	2 (100.0)	4 (100.0)	8 (100.0)	9 (100.0)	0.39
**Site 5** (n=17)	15 (88.2)	1 (100.0)	1 (50.0)	2 (100.0)	4 (100.0)	4 (100.0)	0 (0.0)	3 (100.0)	0.82
**Site 6** (n=51)	41 (80.4)	7 (87.5)	9 (81.8)	15 (88.2)	3 (60.0)	4 (100.0)	2 (50.0)	1 (50.0)	0.16
**Site 7** (n=154)	117 (75.9)	4 (50.0)	14 (53.9)	12 (70.6)	20 (83.3)	24 (80.0)	22 (91.7)	21 (84.0)	**<0.001**
**Site 8** (n=54)	8 (14.8)	0 (0.0)	0 (0.0)	0 (0.0)	0 (0.0)	0 (0.0)	5 (62.5)	3 (75.0)	**<0.001**

© 2025 Kennedy ED et al. *JAMA Network Open*.

Supplemental Tables: Uptake of Process Measures

Panel D: Uptake of Process Measures: MCC Synoptic Report

Sites	Overall	Time 1	Time 2	Time 3	Time 4	Time 5	Time 6	Time 7	p-value
**Site 1** (n=95)	78 (82.1)	2 (66.7)	2 (40.0)	6 (33.0)	15 (93.8)	19 (100)	19 (100)	15 (100)	**<0.001**
**Site 2** (n=30)	3 (10.0)	0 (0.0)	1 (11.1)	2 (33.3)	0 (0.0)	0 (0.0)	0 (0.0)	0 (0.0)	0.26
**Site 3** (n=7)	1 (14.3)	0 (0.0)	0 (0.0)	0 (0.0)	0 (0.0)	0 (0.0)	0 (0.0)	1 (50.0)	0.43
**Site 4** (n=44)	20 (45.5)	1 (16.7)	4 (50.0)	2 (28.6)	2 (100.0)	2 (50.0)	4 (50.0)	5 (55.6)	0.22
**Site 5** (n=15)	12 (80.0)	0 (0.0)	0 (0.0)	1 (50.0)	4 (100.0)	4 (100.0)	0 (0.0)	3 (100.0)	**0.007**
**Site 6** (n=41)	32 (78.1)	3 (42.9)	6 (66.7)	13 (86.7)	3 (100.0)	4 (100.0)	2 (100.0)	1 (100.0)	**0.006**
**Site 7** (n=117)	29 (24.8)	1 (25.0)	0 (0.0)	0 (0.0)	1 (5.0)	1 (4.2)	7 (31.8)	19 (90.5)	**<0.001**
**Site 8** (n=8)	8 (100.0)	0 (0.0)	0 (0.0)	0 (0.0)	0 (0.0)	0 (0.0)	5 (100.0)	3 (100.0)	**-**

Panel E: Uptake of Process Measures: Pre-treatment MRI

Sites	Overall	Time 1	Time 2	Time 3	Time 4	Time 5	Time 6	Time 7	p-value
**Site 1** (n=130)	95 (73.1)	11 (57.9)	10 (58.9)	17 (68.0)	14 (87.5)	17 (89.5)	15 (78.9)	11 (73.3)	0.04
**Site 2** (n=48)	39 (81.3)	9 (90.0)	11 (68.8)	5 (71.4)	2 (100.0)	3 (100.0)	3 (100.0)	6 (85.7)	0.43
**Site 3** (n=92)	78 (84.8)	10 (90.9)	15 (93.8)	4 (80.0)	19 (82.6)	8 (100.0)	10 (83.3)	12 (70.6)	0.12
**Site 4** (n=46)	43 (93.5)	5 (83.3)	8 (88.9)	7 (87.5)	2 (100.0)	4 (100.0)	8 (100.0)	9 (100.0)	0.12
**Site 5** (n=17)	17 (100.0)	1 (100.0)	2 (100.0)	2 (100.0)	4 (100.0)	4 (100.0)	1 (100.0)	3 (100.0)	**-**
**Site 6** (n=51)	48 (94.1)	8 (100.0)	10 (90.9)	17(100.0)	5 (100.0)	4 (100.0)	3 (75.0)	1 (50.0)	**0.04**
**Site 7** (n=154)	131 (85.1)	7 (87.5)	23 (88.5)	12 (70.6)	22 (91.7)	27 (90.0)	20 (83.3)	20 (80.0)	0.72
**Site 8** (n=54)	49 (90.7)	13 (92.9)	11 (84.6)	4 (100.0)	6 (100.0)	4 (80.0)	8 (100.0)	3 (75.0)	0.91

Panel F: Uptake of Process Measures: Synoptic MRI Report

Sites	Overall	Time 1	Time 2	Time 3	Time 4	Time 5	Time 6	Time 7	p-value
**Site 1** (n=95)	47 (49.5)	3 (27.3)	3 (30.0)	6 (35.3)	7 (50.0)	10 (58.8)	11 (73.3)	7 (63.6)	**0.003**
**Site 2** (n=39)	30 (76.9)	4 (44.4)	10 (90.9)	4 (80.0)	0 (0.0)	3 (100.0)	3 (100.0)	6 (100.0)	**0.04**
**Site 3** (n=78)	13 (16.7)	0 (0.0)	2 (13.3)	0 (0.0)	2(10.5)	2 (25.0)	1 (10.0)	6 (50.0)	**0.007**
**Site 4** (n=43)	41 (95.4)	4 (80.0)	8 (100.0)	7 (100.0)	2 (100.0)	4 (100.0)	8 (100.0)	8 (88.9)	1.00
**Site 5** (n=17)	15 (88.2)	1 (100.0)	1 (50.0)	2 (100.0)	3 (75.0)	4 (100.0)	1 (100.0)	3 (100.0)	0.32
**Site 6** (n=48)	32 (66.7)	5 (62.5)	7 (70.0)	11 (64.7)	4 (80.0)	3 (75.0)	1 (33.3)	1 (100.0)	1.00
**Site 7** (n=131)	41 (30.6)	0 (0.0)	1 (4.4)	4 (30.8)	7 (31.8)	6 (22.2)	11 (50.0)	12 (60.0)	**<0.001**
**Site 8** (n=49)	34 (69.4)	12 (92.3)	7 (63.6)	3 (75.0)	3 (50.0)	3 (75.0)	5 (62.5)	1 (33.3)	0.07

© 2025 Kennedy ED et al. *JAMA Network Open.*

Panel G: Uptake of Process Measures: Radiation Oncology Peer Review

Sites	Overall	Time 1	Time 2	Time 3	Time 4	Time 5	Time 6	Time 7	p-value
**Site 1** (n=80)	2 (2.5)	0 (0.0)	1 (10.0)	0 (0.0)	1 (10.0)	0 (0.0)	0 (0.0)	0 (0.0)	0.73
**Site 2** (n=16)	9 (56.3)	6 (100.0)	2 (40.0)	0 (0.0)	1 (100.0)	0 (0.0)	0 (0.0)	0 (0.0)	**0.03**
**Site 3** (n=61)	3 (4.9)	1 (11.1)	1 (14.3)	0 (0.0)	1 (6.3)	0 (0.0)	0 (0.0)	0 (0.0)	0.11
**Site 4** (n=28)	13 (46.4)	1 (25.0)	2 (28.6)	2 (100.0)	0 (0.0)	2 (50.0)	2 (40.0)	4 (80.0)	0.15
**Site 5** (n=5)	4 (80.0)	0 (0.0)	1 (50.0)	1 (100.0)	1 (100.0)	0 (0.0)	0 (0.0)	1 (100.0)	0.60
**Site 6** (n=31)	29 (93.6)	4 (66.7)	6 (100.0)	10(100.0)	4 (100.0)	3 (100.0)	1 (100.0)	1 (100.0)	0.09
**Site 7** (n=67)	1 (1.4)	0 (0.0)	0 (0.0)	0 (0.0)	0 (0.0)	0 (0.0)	1 (7.7)	0 (0.0)	0.53
**Site 8** (n=28)	27 (96.4)	9 (100.0)	5 (100.0)	3 (100.0)	3 (75.0)	2 (100.0)	5 (100.0)	0 (0.0)	0.89

Panel H: Uptake of Process Measures: Radiation Oncology Checklist

Sites	Overall	Time 1	Time 2	Time 3	Time 4	Time 5	Time 6	Time 7	p-value
**Site 1** (n=80)	4 (5.0)	0 (0.0)	0 (0.0)	0 (0.0)	0 (0.0)	0 (0.0)	2 (18.1)	2 (33.3)	**0.002**
**Site 2** (n=16)	8 (50.0)	4 (66.7)	3 (60.0)	0 (0.0)	1 (100.0)	0 (0.0)	0 (0.0)	0 (0.0)	0.31
**Site 3** (n=61)	3 (4.9)	1 (11.1)	1 (14.3)	0 (0.0)	1 (6.3)	0 (0.0)	0 (0.0)	0 (0.0)	0.11
**Site 4** (n=28)	13 (46.4)	1 (25.0)	2 (28.6)	2 (100.0)	0 (0.0)	2 (50.0)	2 (40.0)	4 (80.0)	0.15
**Site 5** (n=5)	4 (80.0)	0 (0.0)	1 (50.0)	1 (100.0)	1 (100.0)	0 (0.0)	0 (0.0)	1 (100.0)	0.60
**Site 6** (n=31)	30 (96.8)	5 (83.3)	6 (100.0)	10(100.0)	4 (100.0)	3 (100.0)	1 (100.0)	1 (100.0)	0.35
**Site 7** (n=67)	3 (4.5)	0 (0.0)	0 (0.0)	0 (0.0)	2 (18.1)	0 (0.0)	1 (7.7)	0 (0.0)	0.75
**Site 8** (n=28)	6 (21.4)	0 (0.0)	0 (0.0)	3 (100.0)	2 (50.0)	1 (50.0)	0 (0.0)	0 (0.0)	0.41

Panel I: Uptake of Process Measures: Pre-op Stoma Site Marking

Sites	Overall	Time 1	Time 2	Time 3	Time 4	Time 5	Time 6	Time 7	p-value
**Site 1** (n=130)	90 (76.3)	10 (55.6)	11 (73.3)	18 (85.7)	9 (69.2)	12 (66.7)	17 (94.4)	13 (86.7)	**0.03**
**Site 2** (n=48)	19 (48.7)	3 (33.3)	2 (16.7)	4 (80.0)	2 (100.0)	1 (50.0)	2 (66.7)	5 (83.3)	**0.009**
**Site 3** (n=92)	11 (12.0)	0 (0.0)	0 (0.0)	0 (0.0)	2 (8.7)	2 (25.0)	2 (16.7)	5 (29.4)	**0.002**
**Site 4** (n=46)	41 (95.3)	5 (100.0)	6 (75.0)	7 (100.0)	2 (100.0)	4 (100.0)	8 (100.0)	9 (100.0)	0.25
**Site 5** (n=17)	13 (76.5)	1 (100.0)	2 (100.0)	2 (100.0)	3 (75.0)	2 (50.0)	0 (0.0)	3 (100.0)	0.94
**Site 6** (n=51)	42. (82.4)	8 (100.0)	8 (72.7)	15 (88.2)	5 (100.0)	4 (100.0)	1 (33.3)	1 (50.0)	0.11
**Site 7** (n=154)	151 (98.1)	8 (100.0)	26 (100.0)	16 (94.1)	24(100.0)	28 (93.3)	24 (100.0)	25 (100.0)	1.00
**Site 8** (n=54)	50 (92.6)	14(100.0)	9 (69.2)	4 (100.0)	6 (100.0)	5 (100.0)	8 (100.0)	4 (100.0)	0.80

© 2025 Kennedy ED et al. *JAMA Network Open*.

Supplemental Tables: Uptake of Process Measures

Panel J: Uptake of Process Measures: Synoptic OR Report

Sites	Overall	Time 1	Time 2	Time 3	Time 4	Time 5	Time 6	Time 7	p-value
**Site 1** (n=130)	95 (73.1)	13 (68.4)	11 (64.7)	18 (72.0)	15 (93.8)	13 (68.4)	14 (73.7)	11 (73.3)	0.61
**Site 2** (n=48)	19 (39.6)	3 (30.0)	2 (12.5)	4 (57.1)	0 (0.0)	2 (66.7)	3 (100.0)	5 (71.4)	**0.002**
**Site 3** (n=92)	18 (19.6)	0 (0.0)	1 (6.3)	0 (0.0)	3 (13.1)	2 (25.0)	3 (25.0)	9 (52.9)	**<0.001**
**Site 4** (n=46)	23 (50.0)	1 (16.7)	1 (11.1)	5 (62.5)	0 (0.0)	2 (50.0)	5 (62.5)	9 (100.0)	**0.002**
**Site 5** (n=17)	7 (41.2)	1 (100.0)	0 (0.0)	1 (50.0)	3 (75.0)	2 (50.0)	0 (0.0)	0 (0.0)	**0.28**
**Site 6** (n=51)	27 (52.9)	7 (87.5)	5 (45.5)	5 (29.4)	1 (20.0)	3 (75.0)	4 (100.0)	2 (100.0)	0.44
**Site 7** (n=154)	136 (88.3)	7 (87.5)	24 (92.3)	16 (94.1)	19 (79.2)	28 (93.3)	20 (83.3)	22 (88.0)	0.64
**Site 8** (n=54)	25 (46.3)	4 (28.6)	2 (15.4)	3 (75.0)	4 (66.7)	3 (60.0)	6 (75.0)	3 (75.0)	**0.002**

Panel K: Uptake of Process Measures: CAP Checklist

Sites	Overall	Time 1	Time 2	Time 3	Time 4	Time 5	Time 6	Time 7	p-value
**Site 1** (n=130)	111 (85.4)	12 (63.2)	16 (94.1)	23 (92.0)	14 (87.5)	15 (78.9)	19 (100.0)	12 (80.0)	0.23
**Site 2** (n=48)	41 (85.5)	9 (90.0)	13 (81.3)	6 (85.7)	0 (0.0)	3 (100.0)	3 (100.0)	7 (100.0)	0.45
**Site 3** (n=92)	81 (88.0)	11(100.0)	14 (87.5)	4 (80.0)	21 (91.3)	7 (87.5)	11 (91.7)	13 (76.5)	0.21
**Site 4** (n=46)	46 (100.0)	6 (100.0)	9 (100.0)	8 (100.0)	2 (100.0)	4 (100.0)	8 (100.0)	9 (100.0)	**-**
**Site 5** (n=17)	17 (100.0)	1 (100.0)	2 (100.0)	2 (100.0)	4 (100.0)	4 (100.0)	1 (100.0)	3 (100.0)	**-**
**Site 6** (n=51)	48 (94.1)	6 (75.0)	11 (100.0)	16 (94.1)	5 (100.0)	4 (100.0)	4 (100.0)	2 (100.0)	0.14
**Site 7** (n=154)	151 (98.1)	6 (75.0)	25 (96.2)	17(100.0)	24(100.0)	30(100.0)	24(100.0)	25(100.0)	**0.001**
**Site 8** (n=54)	54(100.0)	14(100.0)	13(100.0)	4(100.0)	6(100.0)	5(100.0)	8(100.0)	4(100.0)	**-**

Panel L: Uptake of Process Measures: Quirke Method

Sites	Overall	Time 1	Time 2	Time 3	Time 4	Time 5	Time 6	Time 7	p-value
**Site 1** (n=130)	106 (81.5)	10 (52.6)	11 (64.7)	24 (96.0)	15 (93.8)	14 (73.7)	17 (89.5)	15 (100)	**0.001**
**Site 2** (n=48)	37 (77.1)	5 (50.0)	13 (81.3)	6 (85.7)	1 (50.0)	2 (66.7)	3 (100.0)	7 (100.0)	**0.048**
**Site 3** (n=92)	8 (8.7)	0 (0.0)	0 (0.0)	0 (0.0)	7 (30.4)	1 (12.5)	0 (0.0)	0 (0.0)	0.98
**Site 4** (n=46)	46 (100.0)	6 (100.0)	9 (100.0)	8 (100.0)	2 (100.0)	4 (100.0)	8 (100.0)	9 (100.0)	**-**
**Site 5** (n=17)	15 (88.2)	1 (100.0)	2 (100.0)	2 (100.0)	4 (100.0)	4 (100.0)	1 (100.0)	1 (33.3)	**0.04**
**Site 6** (n=51)	46 (90.2)	8 (100.0)	8 (72.7)	16 (94.1)	4 (80.0)	4 (100.0)	4 (100.0)	2 (100.0)	0.49
**Site 7** (n=154)	154 (100.0)	8 (100.0)	26 (100.0)	17(100.0)	24(100.0)	30(100.0)	24(100.0)	25(100.0)	**-**
**Site 8** (n=54)	51 (94.4)	13 (92.9)	13(100.0)	4(100.0)	6(100.0)	4(80.0)	7(87.5)	4(100.0)	0.58

© 2025 Kennedy ED et al. *JAMA Network Open*.
