# Supplementary material for: Knowledge Translation Interventions to Address Gaps in Rectal Cancer Care
Source: JAMA Netw Open. 2025 Feb 17;8(2):e2461047. doi: 10.1001/jamanetworkopen.2024.61047 (PMC11833516; doi:10.1001/jamanetworkopen.2024.61047)
Supplement: Supplement 2. — Data Sharing Statement [file jamanetwopen-e2461047-s002.pdf]

Data Sharing Statement

Kennedy E, Pooni A, Schmocker S, et al. Knowledge Translation Interventions to Address Gaps in Rectal Cancer Care. *JAMA Netw Open*. Published online February 17, 2025. doi:10.1001/jamanetworkopen.2024.61047

Data
Data available: No

Additional Information
Explanation for why data not available: Data are available from the PI following appropriate institution and ethics approvals.
